# Uncovering the Changing Gene Expression Profile of Honeybee (*Apis mellifera*) Worker Larvae Transplanted to Queen Cells

**DOI:** 10.3389/fgene.2018.00416

**Published:** 2018-10-24

**Authors:** Ling Yin, Kang Wang, Lin Niu, Huanxin Zhang, Yuyong Chen, Ting Ji, Guohong Chen

**Affiliations:** ^1^Jiangsu Agri-animal Husbandry Vocational College, Taizhou, China; ^2^College of Animal Science and Technology, Yangzhou University, Yangzhou, China

**Keywords:** gene expression, honeybee, queen bee, transcriptomics, caste differentiation

## Abstract

The reproductive division of labor, based on caste differentiation in social insects, is of great significance in evolution. Generally, a healthy bee colony consists of a queen and numerous workers and drones. Despite being genetically identical, the queen and workers exhibit striking differences in morphology, behavior, and lifespan. The fertilized eggs and larvae selectively develop into queen and worker bees depending on the local nutrition and environment. Bee worker larvae that are transplanted within 3 days of age to queen cells of a bee colony can develop into queens with mature ovaries. This phenomenon is important to understand the regulatory mechanisms of caste differentiation. In this study, we transplanted worker larvae (*Apis mellifera*) at the age of 1 (L1), 2 (L2), and 3 days (L3) into queen cells until the age of 4 days. Subsequently, genetic changes in these larvae were evaluated. The results revealed that the number of differentially expressed genes (DEGs) in L1 vs. L3 was more than that in L1 vs. L2. Furthermore, many of the genes that were downregulated are mostly involved in metabolism, body development, reproductive ability, and longevity, indicating that these functions decreased with the age of transplantation of the larvae. Moreover, these functions may be critical for worker larvae to undergo the developmental path to become queens. We also found that the DEGs of L1 vs. L2 and L1 vs. L3 were enriched in the MAPK, FoxO, mTOR, Wnt, TGF-beta Hedgehog Toll and Imd, and Hippo signaling pathways. Gene ontology analysis indicated that some genes are simultaneously involved in different biological pathways; through these genes, the pathways formed a mutual regulatory network. Casein kinase 1 (CK 1) was predicted to participate in the FoxO, Wnt, Hedgehog, and Hippo signaling pathways. The results suggest that these pathways cross talked through the network to modify the development of larvae and that CK 1 is an important liaison. The results provide valuable information regarding the regulatory mechanism of environmental factors affecting queen development, thus, amplifying the understanding of caste differentiation in bees.

## Introduction

The efficient and refined division of labor among social insects, such as termites, wasps, ants, and bees, plays a key role in their ecological success (Wilson, 1985). A normal honeybee swarm is usually composed of three types of individuals, namely diploid females that include a queen and worker bees and haploid males (drones). Marked differences exist between the queen and worker bees in terms of morphology, behavior, reproductive ability, function, and life span (Wilson, 1971). This phenomenon in social insects is known as caste determination and is affected by environmental factors (West-Eberhard, 1989; Evans and Wheeler, 2001). Although both the queen and worker bees develop from fertilized eggs, the larvae gradually develop to a queen and worker bees, depending on the environment (Cristino et al., 2006). Plasticity is one of the key characteristics of the division of labor in social insects (Robinson, 1992), and it regulates the process of caste determination. Caste determination is not usually inherited but is mediated by external factors.

Fertilized eggs and larvae selectively develop into a queen and worker bees depending on local nutrition and environmental factors. If worker larvae of *Apis mellifera* are transplanted within 3 days of age to the queen cells of a bee colony and incubated, they can grow and develop into queen bees with mature ovaries (He et al., 2016). In China, a standard practice in commercial beekeeping is to raise queens by transplanting eggs or young larvae into artificial queen cells (Doolittle, 1888; Büchler et al., 2013), triggering the development of a queen that produces royal jelly (Zhijiang, 2013). In commercial queen rearing practices, there is variation in the age at which worker larvae are transplanted to queen cells to be raised as queens. Some reports have shown that queens reared from older worker larvae have decreased body sizes, a smaller spermatheca, and fewer ovarioles compared with those of the queens reared from younger worker larvae (Rangel et al., 2012; Qian et al., 2017). Therefore, the queens reared from older worker larvae have decreased reproductive ability, and the colony produces a significantly smaller worker comb and drone comb and has lower stored food. The quality of the queen is the most important economic trait of a colony. Although it is well known that environmental factors affect the quality of a queen, the underlying regulatory mechanism is unclear. In this study, the transcriptomes of 4-day-old larvae transferred at different ages to the queen cell were analyzed by RNA-sequencing (RNA-Seq) to monitor changes in the expression profile of larvae that developed into queens possessing different qualities. The differential regulation of development led to caste determination, eventually leading to reproductive division of labor; thus, our study provides valuable information on the regulatory mechanisms of environmental factors affecting the quality of a queen and amplifying the understanding of caste differentiation in bees.

## Materials and Methods

### Honeybee Larvae and Experimental Procedures

To minimize noise in the genetic background, *A. mellifera carnica* bees were derived from a single drone-inseminated queen. The colonies were raised at the Honeybee Research Institute of Yangzhou University in Yangzhou, China.

**FIGURE 1:**
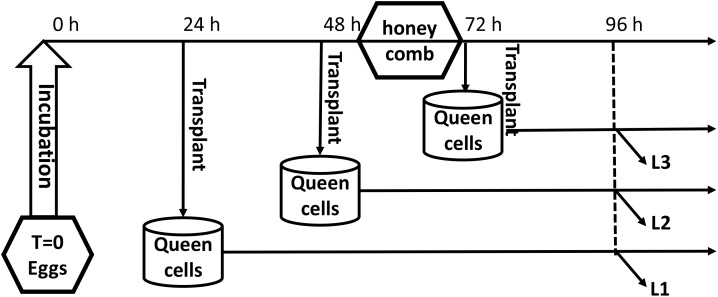
The experimental design.

The queens were confined for 6 h in a blanket honeycomb to lay eggs. Worker larvae aged 1, 2, and 3 days that developed from the eggs, which the queen laid in worker cells, were transplanted to the queen cells and reared to the age of 4 days; the three groups were named L1, L2, and L3, respectively. Each group had three biological replicates, and each replicate included three larvae. The experimental design is shown in Figure 1. All samples were immediately flash-frozen in liquid nitrogen. RNA from each sample was extracted and subjected to RNA-Seq analysis at Shanghai OE Biotech Company.

### RNA Isolation, Library Preparation, and Sequencing

Total RNA was extracted from three larvae using the mirVana^TM^ miRNA Isolation Kit (Ambion-1561) and was pooled by following the protocol of the manufacturer. The integrity of RNA was evaluated using the Bioanalyzer 2100 RNA-6000 Nano Kit (Agilent Technologies). Samples with an RNA integrity number (RIN) ≥ 7 were analyzed further. Complementary DNA (cDNA) libraries were constructed from mRNA that was isolated using the TruSeq Stranded Total RNA Sample Prep Kit, based on enrichment with oligo (dT) magnetic beads and fragmentation (approximately 200–700 nucleotides) in fragmentation buffer. Briefly, random hexamer primers were used to amplify and prepare the library. Double-stranded cDNA products were purified using the QiaQuick PCR Extraction Kit (Qiagen) and eluted in EB buffer for end repair and poly (A) addition. These libraries were then sequenced on a HiSeqTM 2500 instrument (Illumina), and 150-bp paired-end reads were generated.

### Mapping Reads to the Predicted Coding Sequence (CDS) of Genes in the Reference Genome

The raw reads of all nine samples studied were processed using the Trimmomatic software (Langmead and Salzberg, 2012). Reads containing poly-N regions and low-quality reads (defined as 50% of bases in a read with a quality value ≤ 5) were removed to obtain clean reads. The clean reads were then mapped to the predicted coding sequences (CDSs) of the corresponding genes of the reference *A. mellifera* genome, using hisat2 (Roberts and Pachter, 2013).

### Expression Annotation and DEG Analysis

The fragments per kilobase million (FPKM) value of each transcript (protein_coding) was calculated using bowtie2 (Trapnell et al., 2010) and eXpress (Anders and Huber, 2012). Differentially expressed genes (DEGs) were identified using the DESeq functions estimateSizeFactors and nbinomTest (Florea et al., 2013). A *P*-value < 0.05 and a fold change > 2 or fold change < 0.5 was set as the threshold for significant differential expression. Hierarchical cluster analysis of the DEGs was performed to explore the expression patterns of the transcripts.

### Functional Analysis of the DEGs

All identified DEGs were matched to the gene ontology (GO) terms (Ashburner et al., 2000) using BLAST2GO (Conesa et al., 2005) by following the standard procedure to perform the Basic Local Alignment Search Tool (BLAST) searches for each gene (BLASTn, DFCI database). Enrichment analysis (Kanehisa et al., 2008) revealed whether the DEGs have related functions.

### Validation of the RNA-Seq Data by Quantitative Real-Time PCR (qRT-PCR)

A total of seven DEGs were randomly selected and examined in qRT-PCR experiments performed with three biological replicates. The reactions were performed using the ABI 7500 system with SYBR Green. β-actin (AB023025), a reference gene, was used as the internal control (Liu et al., 2013). The qRT-PCR data were expressed relative to the expression of β-actin. The sequences of the gene-specific primers are shown in Supplementary File S1.

## Results

### RNA-Seq and Analysis of the Raw Data

After filtering the adaptor sequences (the regions containing poly-N and low-quality sequences), over 86.36 million clean reads were produced in each library. An overview of the sequencing statistics is displayed in Table 1. The percent of clean reads among the raw reads in each library ranged from 96.19 to 97.05%, and Q30 (percent of bases with a Phred score > 30 among the raw bases) was more than 93.34% (Table 1), suggesting that the high-quality RNA-Seq data obtained could be used for further analysis. An average of 68.41 million reads per sample was mapped to the predicted CDS of the corresponding genes in the honeybee (*A. mellifera*) genome (64.93–71.66 million). Of the total reads, the rate of reads that matched was > 75% (Table 2). The correlation value of the three biological replicates for each sample was > 0.99 (*R*^2^ > 0.99) based on the values (Supplementary Figure S1). The reported sequencing data has been approved and assigned to the Sequence Read Archive (SRA) database (SRA accession number: SRP158315).

**Table 1:** Throughput and quality of RNA sequencing data.

Sample	Raw reads	Clean reads	Percent of clean reads	GC percent (%)	Q30 percent (%)
Sample_L1_1	89589486	86769356	0.968521641	41.50%	93.94%
Sample_L1_2	86459394	83172042	0.961978082	41.50%	93.34%
Sample_L1_3	87437832	84118264	0.962035106	41.00%	93.39%
Sample_L2_1	89173290	86269582	0.967437469	40.00%	94.07%
Sample_L2_2	87318644	84739966	0.970468185	41.00%	94.36%
Sample_L2_3	86361798	83220106	0.963621739	40.00%	93.68%
Sample_L3_1	87818532	84839102	0.966072879	41.00%	93.93%
Sample_L3_2	89477462	86620466	0.968070216	41.00%	94.13%
Sample_L3_3	89125256	86298048	0.968278262	41.00%	94.17%


**Table 2:** Summary of reads mapped to the reference genome of *Apis mellifera.*

Sample	Sample_L1_2	Sample_L1_1	Sample_L1_3	Sample_L2_2	Sample_L2_1	Sample_L3_3	Sample_L2_3	Sample_L3_1	Sample_L3_2
Total reads	83172042	86769356	84118264	84739966	86269582	86298048	83220106	84839102	86620466
Total mapped reads	69676988 (83.77%)	70823525 (81.62%)	67621141 (80.39%)	68114357 (80.38%)	71661953 (83.07%)	68670887 (79.57%)	65399143 (78.59%)	64927181 (76.53%)	68870251 (79.51%)
Multiple mapped	880546 (1.06%)	902330 (1.04%)	848781 (1.01%)	883477 (1.04%)	885178 (1.03%)	909010 (1.05%)	808646 (0.97%)	825601 (0.97%)	914938 (1.06%)
Uniquely mapped	68796442 (82.72%)	69921195 (80.58%)	66772360 (79.38%)	67230880 (79.34%)	70776775 (82.04%)	67761877 (78.52%)	64590497 (77.61%)	64101580 (75.56%)	67955313 (78.45%)
Read-1	34329259 (41.27%)	34873412 (40.19%)	33331119 (39.62%)	33630071 (39.69%)	35429434 (41.07%)	33918281 (39.30%)	32323040 (38.84%)	32070186 (37.80%)	34004794 (39.26%)
Read-2	34467183 (41.44%)	35047783 (40.39%)	33441241 (39.76%)	33600809 (39.65%)	35347341 (40.97%)	33843596 (39.22%)	32267457 (38.77%)	32031394 (37.76%)	33950519 (39.19%)
Reads map to ‘+’	34430320 (41.40%)	34985553 (40.32%)	33391713 (39.70%)	33649542 (39.71%)	35398775 (41.03%)	33894805 (39.28%)	32297706 (38.81%)	32023469 (37.75%)	33984253 (39.23%)
Reads map to ‘-’	34366122 (41.32%)	34935642 (40.26%)	33380647 (39.68%)	33581338 (39.63%)	35378000 (41.01%)	33867072 (39.24%)	32292791 (38.80%)	32078111 (37.81%)	33971060 (39.22%)
Non-splice reads	47803802 (57.48%)	48628225 (56.04%)	45897286 (54.56%)	46439326 (54.80%)	48714867 (56.47%)	46375151 (53.74%)	44519421 (53.50%)	44059225 (51.93%)	46795353 (54.02%)
Splice reads	20992640 (25.24%)	21292970 (24.54%)	20875074 (24.82%)	20791554 (24.54%)	22061908 (25.57%)	21386726 (24.78%)	20071076 (24.12%)	20042355 (23.62%)	21159960 (24.43%)
Reads mapped in proper pairs	65372332 (78.60%)	66310718 (76.42%)	63153412 (75.08%)	63709484 (75.18%)	66844568 (77.48%)	64018970 (74.18%)	60946450 (73.24%)	60559126 (71.38%)	63793796 (73.65%)


**FIGURE 2:**
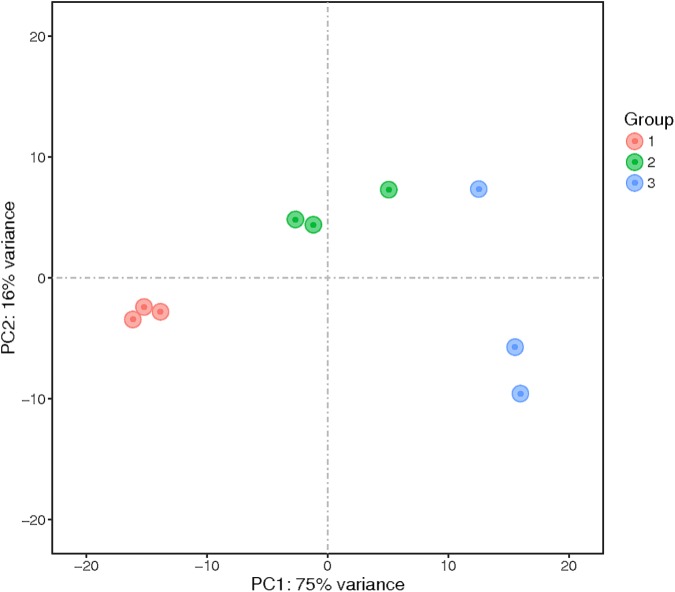
Principal component analysis (PCA) of the transcriptomes of nine samples. The numbers represent the proportion of variance explained by that principal component. The samples represented in different colors were from different groups. PC1 and PC2 represent the top two dimensions of the genes showing differential expression among these samples, accounting for 75% and 16% of the expressed genes, respectively.

Principal component analysis was performed, and the results showed that the samples clustered into three groups (Figure 2). These results indicated sufficient reproducibility and rationality of sampling.

### DEGs in Worker Larvae Transplanted at Different Ages

**FIGURE 3:**
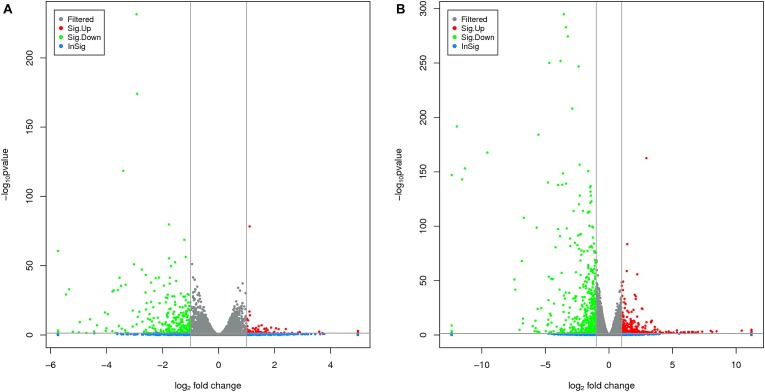
Volcano plots of different differentially expressed genes (DEGs) between two groups. **(A)** The volcano plot of DEGs between L1 vs. L2. **(B)** The volcano plot of DEGs between L1 vs. L3. The horizontal and vertical lines indicate the significance threshold (FDR ≤ 0.05) and 2-fold change threshold (| log 2 Ratio| ≥ 1), respectively. Green dots indicate downregulated genes, black dots indicate genes without differential expression, and red dots indicate upregulated genes.

**FIGURE 4:**
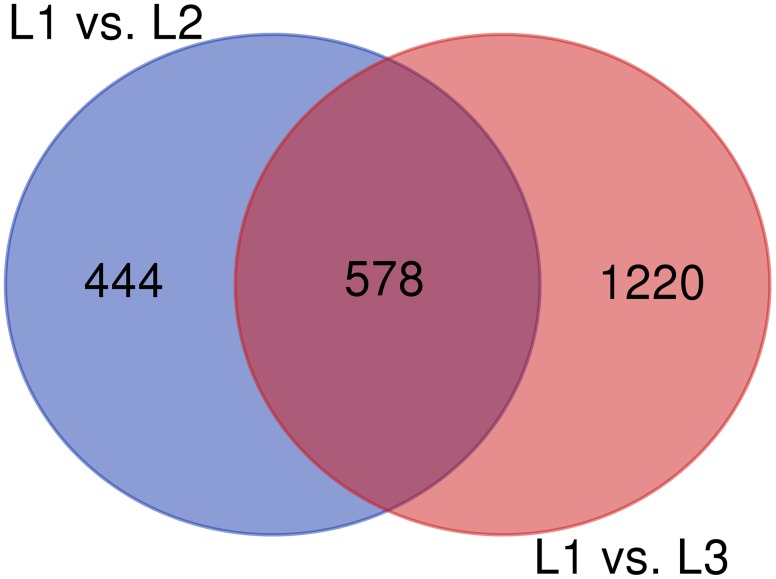
Venn diagram of DEGs among different groups.

The RNA-Seq analysis was performed to compare the gene expression levels between the three groups (L1, L2, and L3). The results showed that the number of DEGs in L1 vs. L3 (1798) was significantly higher than that in L1 vs. L2 (1022). Compared with those in L1, more than 60% of the genes in L2 and L3 were downregulated, and the number of downregulated genes increased with the age of the transplanted worker larva (Figure 3). We observed 578 genes that were either down or upregulated in L2 and L3 of which 413 genes were downregulated and only 165 were upregulated (Figure 4). This finding implied that these shared downregulated genes likely play pivotal roles during larval developmental after transplantation. Among the downregulated genes, a high proportion of genes was involved in metabolism, body development, reproductive ability, and longevity (Supplementary File S2).

### Functional Annotation and Classification

**FIGURE 5:**
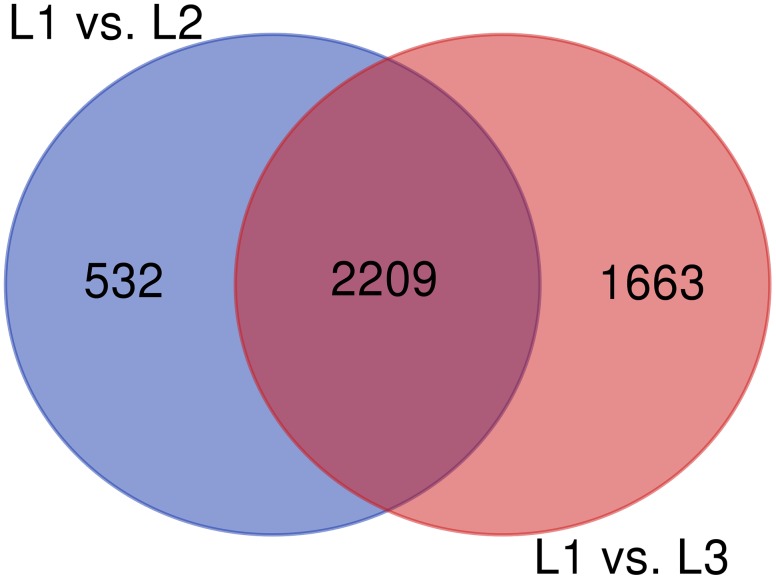
Venn diagram of the Gene ontology (GO) terms among different groups.

**FIGURE 6:**
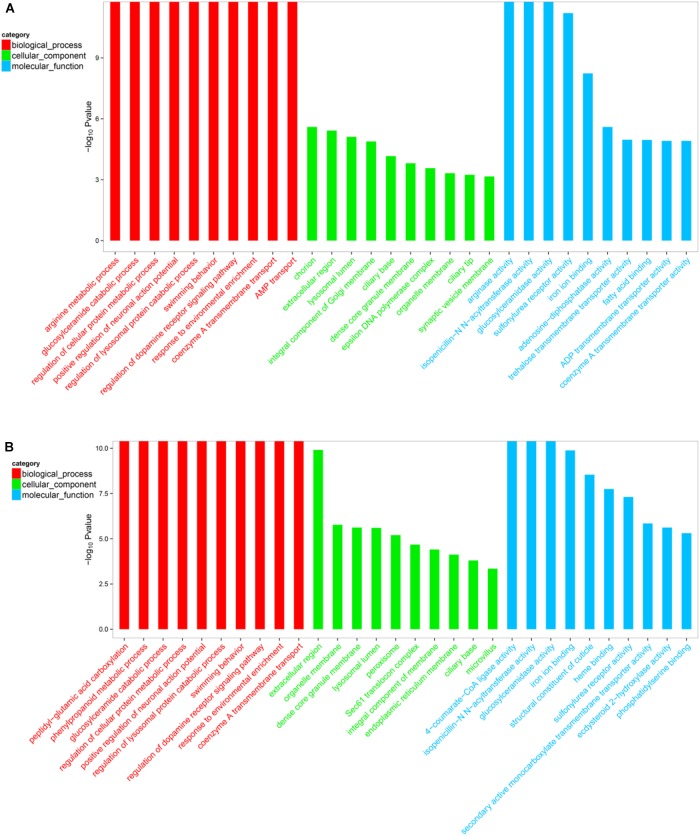
Gene ontology (GO) top-30 enrichment analysis of DEGs. **(A)** GO classification of the DEGs in L1 vs. L2. **(B)** GO classification of the DEGs in L1 vs. L2. The results are grouped into three main categories: biological processes, cellular components, and molecular functions. The X-axis indicates the GO terms, and the Y-axis indicates the percentages of the corresponding genes.

The DEGs within the L1 vs. L2 and L1 vs. L3 groups (*p* < 0.05) were assigned GO terms related to their cellular components, molecular functions, and biological processes. The 2741 and 3872 GO terms were displayed in the Supplementary File S3. Furthermore, GO top-30 enrichment analysis (top 10 enriched genes in terms of molecular function, biological process, and cellular component categories) (Figures 5, 6) revealed that 24 of the top-30 enriched terms were same between the two groups (Figure 6). The results of the GO enrichment analysis showed a similar pattern between L1 vs. L2 and L1 vs. L3.

### Kyoto Encyclopedia of Genes and Genomes (KEGG) Pathway Enrichment Analysis of DEGs

**FIGURE 7:**
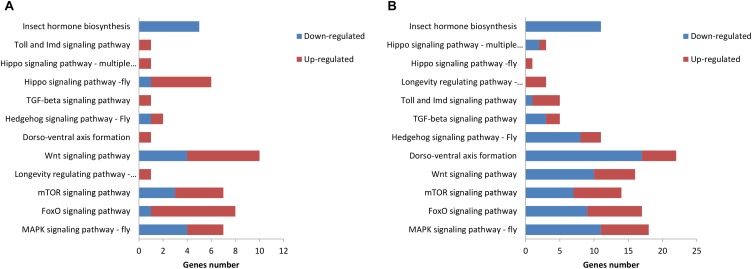
Kyoto Encyclopedia of Genes and Genomes (KEGG) pathway related to caste differentiation. **(A)** KEGG pathway enrichment analysis of DEGs in L1 vs. L2. **(B)** KEGG pathway enrichment analysis of the DEGs in L1 vs. L3.

**FIGURE 8:**
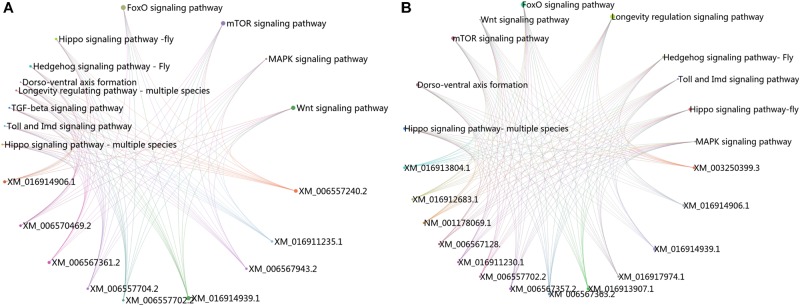
Cross talk between KEGG pathways related to caste differentiation. **(A)** Cross talk between enriched KEGG pathways for the DEGs in L1 vs. L2. **(B)** Cross talk between enriched KEGG pathways for the DEGs in L1 vs. L3.

The KEGG pathway enrichment analysis was conducted for the DEGs within the L1 vs. L2 and L1 vs. L3 groups. A total of 171 DEGs within the L1 vs. L2 group were enriched for 102 KEGG pathways, whereas 363 DEGs within the L1 vs. L3 group were enriched for 112 pathways. The DEGs within the L1 vs. L2 and L1 vs. L3 groups were enriched for 95 of the same pathways (Supplementary File S4) of which 52 pathways were related to synthesis and metabolism, 16 to genetic information processing, 13 to embryonic development, 12 to cellular processes, and 2 to human diseases (Supplementary File S4). Among these pathways, several are thought to be related to caste differentiation (Barchuk et al., 2007; Wheeler et al., 2014; Ashby et al., 2016; Duncan et al., 2016; Fernandez-Nicolas and Belles, 2016), including the MAPK signaling pathway (fly), FoxO signaling pathway, mTOR signaling pathway, longevity regulating pathway (multiple species), Wnt signaling pathway, dorsoventral axis formation, Hedgehog signaling pathway (fly), TGF-beta signaling pathway, Hippo signaling pathway (fly), Hippo signaling pathway (multiple species), Toll and Imd signaling pathway, and insect hormone biosynthesis. The number of DEGs (especially downregulated genes) in these pathways increased with the age of the transplanted worker larva (Figure 7). Further analysis of the genes of several biological pathways showed that some genes are simultaneously involved in multiple biological pathways (Figure 8). Among these genes, transcriptional regulator Myc-B (XM_016914906.1), casein kinase 1 (CK 1)-like (XM_016914939. 1), and S-phase kinase-associated protein 2 (XM_006557702.2) were either upregulated or downregulated in the L2 and L3 groups compared with the L1 group. In particular, CK 1-like participates simultaneously in the Wnt, FoxO, Hedgehog, Hippo, TGF-beta, and longevity regulating pathways and was upregulated in the L2 and L3 groups compared with expression in the L1 group. The transcriptional regulator Myc-B participates in the Wnt, Hippo, and TGF-beta pathways, where S-phase kinase-associated protein 2 participates in FoxO and mTOR pathways simultaneously; both DEGs were downregulated in the L2 and L3 groups when compared with expression in the L1 group.

### Confirmation of the RNA-Seq Data by RT-qPCR

**FIGURE 9:**
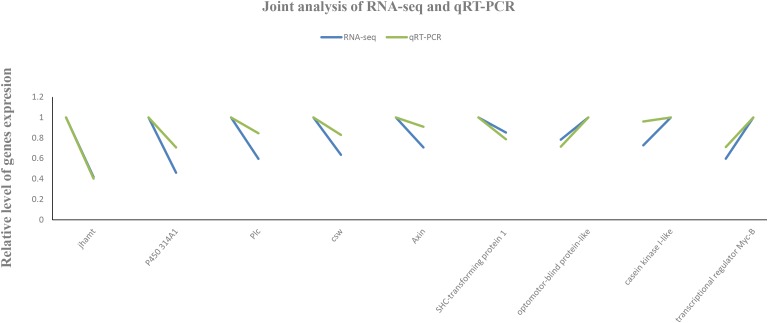
Real-time quantitative PCR verification of the RNA sequencing data.

To validate the accuracy and reproducibility of the transcriptome results, seven DEGs were randomly selected for RT-qPCR verification. Total RNA samples isolated from the L1, L2, and L3 groups were used as templates. The results showed that the expression patterns of candidate genes were consistent with the RNA-Seq data (Figure 9), which confirmed that the measured changes in gene expression detected by RNA-Seq indeed reflected transcriptome differences between the different libraries.

## Discussion

Honeybee is a major model organism among eusocial insects. Theoretically, its complex social behavior can be interpreted to reflect changes in gene expression. The caste differentiation phenomenon of female bees can be traced to DEGs, which determine the developmental fate of a larva. The worker larvae are usually moved to an artificial queen cell to produce royal jelly. Furthermore, it has been found that worker larvae that are less than 3 days old can develop into queens; however, the transplanted older worker larvae develop into individuals of smaller size and lower weight when compared with those of the adult queens (Weaver, 1957; Woyke, 1971; Gilley et al., 2003). Worker larvae that are more than 3 days old when transplanted fail to develop into queens (Weaver, 1957). In this study, we explored genetic changes in 4-day-old worker larvae transplanted into queen cells at the age of 1, 2, and 3 days, which could affect the fate of the worker larvae. The results showed that the downregulated DEGs are mostly involved in metabolism, body development, reproductive ability, and longevity, indicating that these processes in a queen may decrease when larvae are transplanted at increasing ages. These functions may be critical for the ability of worker larvae to develop into queens, and when these functions in larvae decreased, the quality of queens decreased. Previous reports showed that the queen quality and colony productivity decreased depending on the queen rearing method (Woyke, 1971; Thomas, 1998; Rangel et al., 2012). This is likely to be caused by a poor-quality queen, which in turn affects the quality of the swarm. In practice, older worker larvae are chosen for transplantation as they are easier to handle and have a high success rate (Chen, 1989); however, this results in the poor quality of the queen and colony. Therefore, it is preferable to rear queens from younger larvae to achieve better outcomes in terms of queen performance and colony function.

Approximately 400 years ago, people realized that the differentiation into queen and worker bees was due to the different food fed to larvae. Later studies showed that this is due to the differences in both the quantity and quality of food (Page and Peng, 2001), among which the royal jelly plays a key role in caste differentiation. Throughout the larval period, the queen larvae consume substantial amounts of royal jelly, but worker larvae receive plenty of royal jelly only for the first 3 days, after which the food of the worker larvae is switched to a mixture of royal jelly, honey, and pollen. Studies have shown that there are a few differences in the main ingredients of the royal jelly provided to the queen larvae and worker larvae during the first 3 days. The food that the queen larvae and worker larvae receive differs significantly in terms of fat, sugar, and protein contents after the first 3 days (Shuel and Dixon, 1960). Therefore, several studies have focused on genetic differences in queen and worker larvae caused by external factors after the age of 3 days (Zhu et al., 2017; Hartfelder et al., 2018), ignoring the influence of the external environment on the development of larvae within the first 3 days. It was found that during queen breeding, the age of the transplanted worker larvae had a significant influence on the development of queen bees, affecting the quality of the queen. Furthermore, the developmental differences occur between queen and worker larvae within the first 3 days of age. Enrichment analysis revealed that 95 pathways were enriched, based on the DEGs in both L1 vs. L2 and L1 vs. L3 groups; the most prominent among these pathways being insect hormone biosynthesis, longevity regulation, dorsoventral axis formation, MAPK, FoxO, mTOR, Hedgehog, TGF-β, Wnt, Hippo, and Toll and Imd signaling pathways.

Hormones play a key role in the caste differentiation of bees. During the developmental stage, two increases in the juvenile hormone (JH) titer occur; the JH titers of queen larvae are significantly higher than those of the worker larvae. This phenomenon is closely related to the development of the ovaries. During the first increase (occurring in the first 5 days), there is a threshold of JH titer; if at this critical period, the larvae JH titer exceeds the threshold, then the ovaries are well developed (Barchuk et al., 2007). Furthermore, Qian et al. (2017) showed that the size of the ovaries decreased when the larvae were transplanted at an older age. In this study, the DEGs were enriched for the insect hormone biosynthesis pathway and were downregulated with the increase in transplanted age, indicating that with transplanted age, hormone synthesis may be subdued. Moreover, 4 days of age is a key period for caste differentiation of the larvae (Hartfelder and Engels, 1998); therefore, a decrease in the JH titer inevitably affects the development of the ovaries, eventually decreasing the queen quality. Previous data also showed that the Hippo, Wnt, TGF-β, and notch signaling pathways together influence the organ size of fruit flies (Barry and Camargo, 2013), which supports the possibility that the development of queen ovaries may be subdued with an increase in the transplanted age. Further, TOR plays a key role in the bidirectional development of honeybees (Patel et al., 2007), and the Wnt, Hippo, notch, MAPK, and TOR signaling pathways were all involved in the caste differentiation of bees (Wheeler et al., 2014; Ashby et al., 2016); caste differentiation is closely related to the larval development state. In addition, the FoxO signaling, dorso-ventral axis formation, Hedgehog, and TGF-β signaling pathways were found to jointly affect the growth of embryo (Duncan et al., 2016; Fernandez-Nicolas and Belles, 2016). The longevity regulating pathway and Toll and Imd signaling pathway can affect the lifespan and immune function of the larvae, which is an important characteristic that differs between queen and worker bees. All the above-mentioned pathways were enriched in this study, indicating that the developmental direction of worker larvae changed by differing degrees due to the changes in activation of these biological pathways. We further analyzed these biological pathways and found that some genes are involved in several biological pathways simultaneously and that these pathways form a mutual regulatory network through these genes. Casein kinase 1 participated in four pathways, namely the FoxO, Wnt, Hedgehog, and Hippo signaling pathways. It has been proven to be involved in the regulation of mammalian cell proliferation and programmed cell death process (Rana et al., 2004; Wang et al., 2013). Transcriptional regulator Myc-B and S-phase kinase-associated protein 2 also participate in the regulation of mammalian cell proliferation (Kim et al., 2003; Bretones et al., 2015), and S-phase kinase-associated protein 2 was associated with ovarian development in mice (Fotovati et al., 2011). Thus, decreased cell proliferation induces decreased body size, a smaller spermatheca, and fewer ovarioles in queens reared from older larvae. The results suggest that these pathways cross talked through the network to modify the developmental pathway of larvae; CK 1 is an important liaison. The results provide valuable information on the regulatory mechanism of environmental factors affecting the queen quality, which amplifies the understanding of caste differentiation in bees.

## Ethics Statement

This study was carried out in accordance with the recommendations of ’Animal Welfare Guidelines of Jiangsu Agri-animal Husbandry Vocational College, animal welfare committee of Jiangsu Agri-animal Husbandry Vocational College’. The protocol was approved by the ’animal welfare committee of Jiangsu Agri-animal Husbandry Vocational College’.

## Author Contributions

LY designed the study and carried out the data analysis. KW participated in drafting the manuscript. LN, HXZ, and YYC participated in sample collection. TJ and GHC provided advice on data analysis and helped draft the manuscript.

## Conflict of Interest Statement

The authors declare that the research was conducted in the absence of any commercial or financial relationships that could be construed as a potential conflict of interest.

## Supplementary Material

FIGURE S1 Correlation values of the three biological replicates of each sample. The correlation coefficients between the samples were obtained according to the mRNA expression, the closer the correlation coefficient is to 1, the more similar the expression pattern between samples is. The abscissa denotes the sample names, and the ordinate denotes the corresponding sample names.

FILE S1 Primer sequences of DEGs identified in various pathways in RNASeq for qRT-PCR validation.

FILE S2 The common DEGs in L1 vs. L2 and L1 vs. L3. The information of the DEGs both up/down regulated in L2 and L3 compared with L1, including the change-fold, ID, and the product of the DEGs.

FILE S3 GO enrichment analysis of DEGs. The GO terms of molecular function, biological process, and cellular component categories enriched by DEGs in L1 vs. L2 and L1 vs. L3.

FILE S4 The common pathways in L1 vs. L2 and L1 vs. L3. The information of the pathways enriched by DEGs in L1 vs. L2 as well as DEGs in L1 vs. L3, including the enrichment score, functional category and DEGs’ ID.
