# Supplementary figures and images for: Uncovering the Changing Gene Expression Profile of Honeybee (Apis mellifera) Worker Larvae Transplanted to Queen Cells
Source: Front Genet. 2018 Oct 24;9:416. doi: 10.3389/fgene.2018.00416 (PMC6207841; doi:10.3389/fgene.2018.00416)

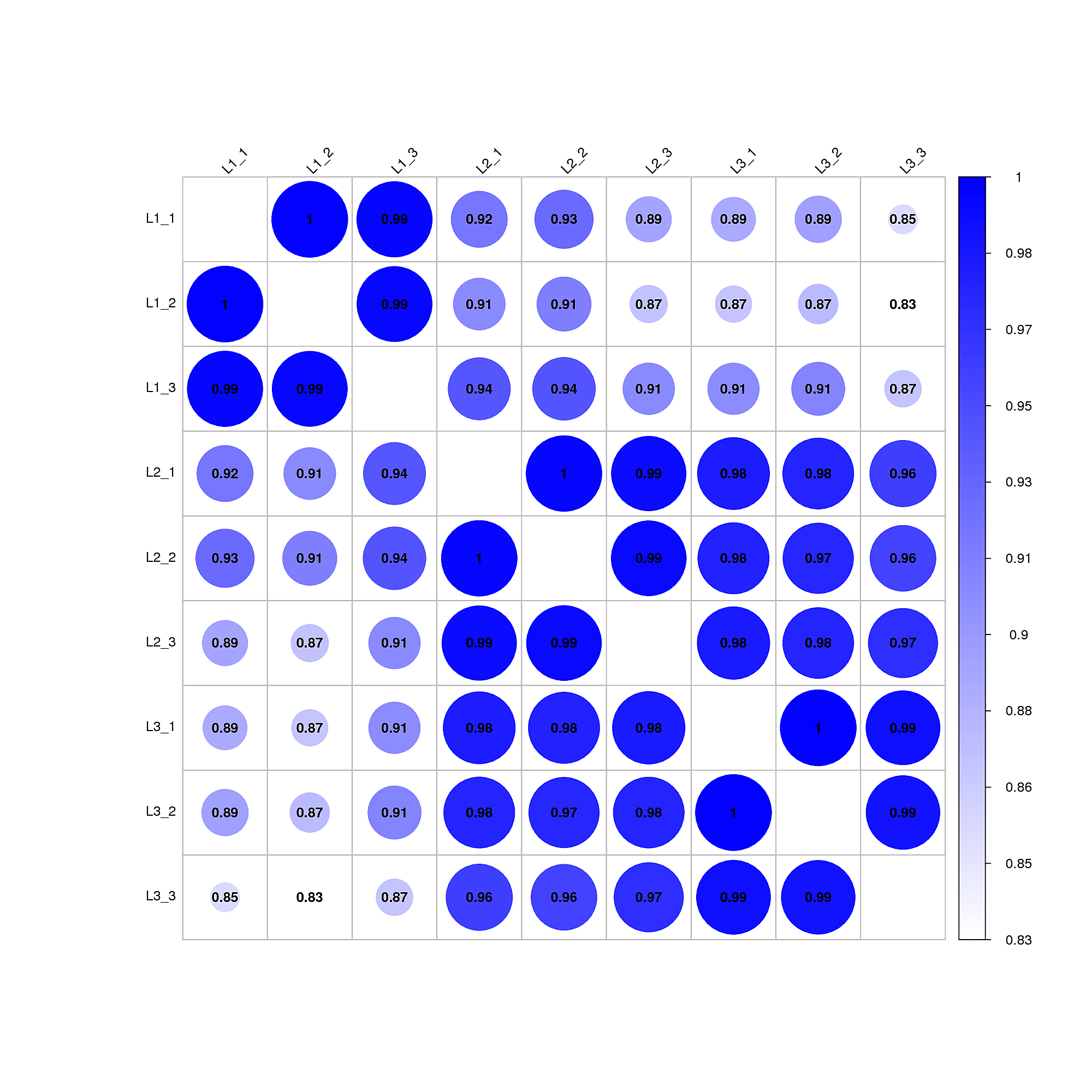

Supplement: FIGURE S1 — Correlation values of the three biological replicates of each sample. The correlation coefficients between the samples were obtained according to the mRNA expression, the closer the correlation coefficient is to 1, the more similar the expression pattern between samples is. The abscissa denotes the sample names, and the ordinate denotes the corresponding sample names. [file Image_1.TIFF]
